# Variation in *DNAH1* may contribute to primary ciliary dyskinesia

**DOI:** 10.1186/s12881-015-0162-5

**Published:** 2015-03-17

**Authors:** Faiqa Imtiaz, Rabab Allam, Khushnooda Ramzan, Moeenaldeen Al-Sayed

**Affiliations:** Department of Genetics, King Faisal Specialist Hospital & Research Centre, PO Box 3354, Riyadh, 11211 Saudi Arabia; Department of Medical Genetics, King Faisal Specialist Hospital & Research Centre, PO Box 3354, Riyadh, 11211 Saudi Arabia

**Keywords:** Primary ciliary dyskinesia, Genome-wide, Axonemal heavy chain, *DNAH*, Whole exome

## Abstract

**Background:**

Primary Ciliary Dyskinesia (PCD) is a genetically heterogeneous ciliopathy caused by ultrastructural defects in ciliary or flagellar structure and is characterized by a number of clinical symptoms including recurrent respiratory infections progressing to permanent lung damage and infertility.

**Case presentation:**

Here we describe our search to delineate the molecular basis in two affected sisters with clinically diagnosed PCD from a consanguineous Saudi Arabian family, in which all known genes have been excluded. A homozygosity mapping-based approach was utilized that ultimately identified one single affected-shared region of homozygosity using 10 additional unaffected family members. A plausible candidate gene was directly sequenced and analyzed for mutations. A novel homozygous missense aberration (p.Lys1154Gln) was identified in both sisters in the *DNAH1* gene that segregated completely with the disease phenotype. Further confirmation of this interesting variant was provided by exome-wide analysis in the proband.

**Conclusion:**

Molecular variation in *DNAH1* may play a role in PCD and its potential contribution should be considered in patients where all known genes are excluded.

**Electronic supplementary material:**

The online version of this article (doi:10.1186/s12881-015-0162-5) contains supplementary material, which is available to authorized users.

## Background

Primary ciliary dyskinesia (PCD, CILD, MIM 244400) is a clinically and genetically heterogeneous disorder of motile cilia dysfunction typically caused by an autosomal recessive mode of inheritance. PCD is characterized by various clinical manifestations, including chronic sinusitis, otitis media and chronic bronchitis leading to permanent lung damage (bronchiectasis). Situs inversus occurs in approximately 50% of PCD cases, a cohort who are specifically sub-diagnosed with Kartagener Syndrome (KS) based on a triad of symptoms [1]. Clinically, PCD affected female patients can present with sub-fertility due to defective oviduct cilia and males can be infertile due to immotile sperm flagella [2]. Occasionally hydrocephalus arises from a decrease of cerebrospinal fluid flow as a consequence of ependymal cilia dysmotility [3,4]. Previous estimates of the worldwide incidence of PCD is 1:15,000-1:30,000 live births but these figures are thought to be underestimated due to under diagnosis [5] due to such reasons as unfamiliarity with the disease and non-distinct signs and symptoms [6]. However, this estimate is reported to be elevated in certain consanguineous and isolated populations [7,8]. Clinically, only half of PCD patients are diagnosed before 5 years of age and diagnosis is often delayed until adolescence, with approximately one-third of these patients at adulthood [6,9,10].

Motile cilia are organelles with a microtubule cytoskeleton core, known as the axenome [11] comprising of nine outer-doublet microtubules, which encircle a central microtubule pair (i.e., 9 + 2 pattern). Outer dynein arms (ODAs) and inner dynein arms (IDAs), which are attached to the peripheral microtubules, provide the power by hydrolyzing ATP to the cilia that generate the force for motility for a number of biological functions and modulate beat frequency [11]. Dynein arms contain varying numbers of heavy (responsible for motor activity), intermediate and light chains [12-15]. Radial spokes and central pair projections are also present in most motile cilia and are further structures known to regulate dynein function [16]. PCD is characterized by ultrastructural defects in the axonemal structure of the 9 + 2 pattern of motile cilia and sperm flagella, for example, a complete or partial loss of inner and/or outer dynein arms, central microtubular abnormalities and defects of radial spokes. Thus, such biological defects are demonstrated clinically by recurrent or chronic sinusitis and bronchiectasis caused by immotile cilia in the upper and lower airways and infertility due to impaired oviduct cilia and sperm flagella [16]. To date, mutations in 20 different genes involved in the structure and function of the motile cilia have been reported to cause PCD, including those that encode the dynein axonemal heavy chain (DNAH) proteins (CILD3 (MIM 608644), caused by mutations in the *DNAH5* gene on chromosome 5p (one of the most common involved in PCD) and CILD7 (MIM 611884), caused by mutations in the *DNAH11* gene on chromosome 7p21. Recently, Ben Khelifa and colleagues [17] reported that homozygous variants in *DNAH1* in five unrelated males were the cause of infertility without any other PCD symptoms, a disorder they proposed to call “multiple morphological anomalies of the flagella (MMAF)”.

## Case presentation

### Methods

#### Research subjects

In this study, because of the genetic heterogeneity of PCD, a homozygosity mapping approach using SNP-microarray analysis was performed in a Saudi Arabian consanguineous family with two clinically affected sisters. This study adhered to institutional guidelines (Research Advisory Council; King Faisal Specialist Hospital & Research Centre) and to the Helsinki Declaration (http://www.wma.net/en/30publications/10policies/b3/).

#### Homozygosity mapping

Genomic DNA from whole blood was isolated from the affected siblings and 10 additional family members using the Gentra DNA Extraction Kit (Qiagen, Germantown, Maryland, USA). Genome-wide genotypes for all individuals were obtained using the Axiom® CEU Human Array (Affymetrix, Inc., Santa Clara, USA) in accordance with the manufacturer’s protocol. Homozygosity mapping was performed using both HomozygosityMapper [18] and AutoSNPa [19] softwares.

#### Sanger sequencing

Intronic primers were designed to flank each of the 77 coding exons of the *DNAH1* (Genbank accession no. NM_015512.4) using the Primer3 v.0.4.0 program (http://bioinfo.ut.ee/primer3-0.4.0/). PCR reactions were typically performed in a 25 μl reaction volume containing standard reagents and 20 ng of genomic DNA. Primer sequences used are available in the supplementary material (Additional file 1: Table S1). Thermocycling consisted of an initial denaturation at 95°C for 10 min followed by 30 cycles of PCR. Each cycle of PCR consisted of denaturation at 94°C for 60 s, annealing at 62–68°C for 60 s and extension at 72°C for 60 s. A final extension step of 10 min at 72°C was added. Sequence analysis was performed using the SeqMan 6.1 module of the Lasergene (DNA Star Inc. WI, USA) software package and then compared to the reference GenBank sequence on which mutation nomenclature was based. Numbering commenced with the A of the ATG initiation codon as +1.

#### Exome sequencing

Commercially available next-generation sequencing Gene panel version DGD_15112013 (Additional file 2: Table S2) was performed on the proband. In addition, whole exome sequencing was also carried out on the proband using the Illumina® HiSeq2000 platform using TruSeqv3 chemistry by preparing and enriching the sample according to the manufacturer’s standard protocol. Concentration of the each library was determined using Agilent’s (Agilent Technologies, Santa Clara, CA, USA) QPCR NGS Library Quantification Kit (G4880A) and the sample was sequenced at a final concentration of 10 nM. Mapping and alignment was performed on read files (Fastq) generated from the sequencing platform via the manufacturer’s proprietary software and using human genome (hg19/b37) using the Burrows-Wheeler Aligner (BWA) package, version 0.6.1 [20]. Further realignment and variant analysis were performed eventually determining SNP novelty against dbSNP (Human Build 135) [21-23]. Variants were annotated with gene and gene function from Ensembl (http://www.ensembl.org/index.html) [24] and further analysis of possible causative variants by filtering the full exome dataset for all deletions, insertions, nonsense and canonical splice-site mutations, as well as missense mutations (with a PhyloP score of >3.5 of the underlying base change) were determined and reported.

## Results

### Clinical report

**Figure 1:**
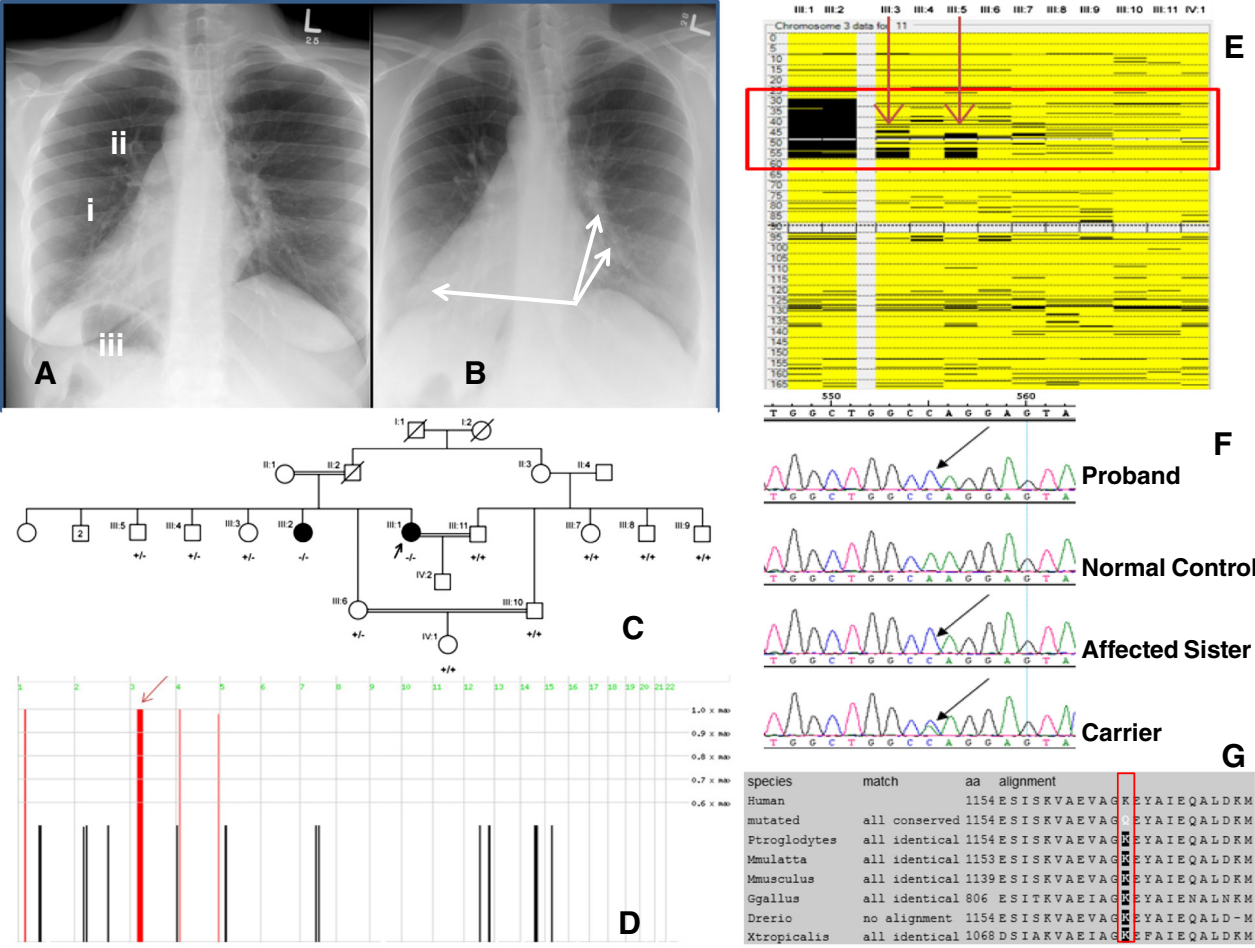
**Clinical and molecular genetic findings.** Panel **A)** AP Chest X-ray of proband indicating (i) dextrocardia with (ii) right-sided aortic arch. The gastric air bubble is also in the right side (iii) that is most likely representing situs inversus. Panel **B)** AP Chest X-ray of proband where arrows are indicating bronchial wall thickening with mild bronchiectasis in the left middle lobe and also in the right lower lobe. Panel **C)** Family pedigree showing the genotypes for the p.Lys1154Gln variation in *DNAH1* for all family members that were sequenced. (+) indicates the wild-type allele and (−) indicates the mutant allele. Arrow indicates the proband. Panel **D)** Results of analysis of genotyping data with Homozygosity Mapper, showing 4 ROHs shared among the affected individuals. The ROH on chromosome 3p23-p14.2 containing *DNAH1* is indicated by an arrow. Panel **E)** Narrowing the single valid ROH by two unaffected family members on chromosome 3 by AutoSNPa analysis. Panel **F)** Sequence chromatogram of exon 20 where the mutant arrow points to the site of the c.3460 A > C transversion and Panel **G)** Multiple protein sequence alignment of DNAH1 orthologs showing complete conservation of the p.Lys1154 residue compared to the altered residue (indicated by red box).

The proband had a previous medical history at 13 years of age with chronic rhinitis, frequent coughing and wheezing, nasal discharge, and was diagnosed with left lung bronchitis. She also presented with a multi-nodular goiter confirmed by fine needle aspiration that revealed a colloid goiter with cystic degeneration. She was treated for hypothyroidism and an adenoidectomy was performed. She was referred to King Faisal Specialist Hospital & Research Centre at the age of 29 for IVF for infertility spanning 5 years. All clinical laboratory tests were within the normal ranges but a chest X-ray and CT scan showed the other two classic KS symptoms in combination with infertility; situs inversus (Figure 1A) and bronchiectasis (Figure 1B). Audiometric evaluation also revealed mild conductive hearing loss. Detailed clinical information was not available for the affected sister of the proband who was diagnosed with KS at a similar age.

### Regions of homozygosity and sanger sequencing

**Figure 2:**
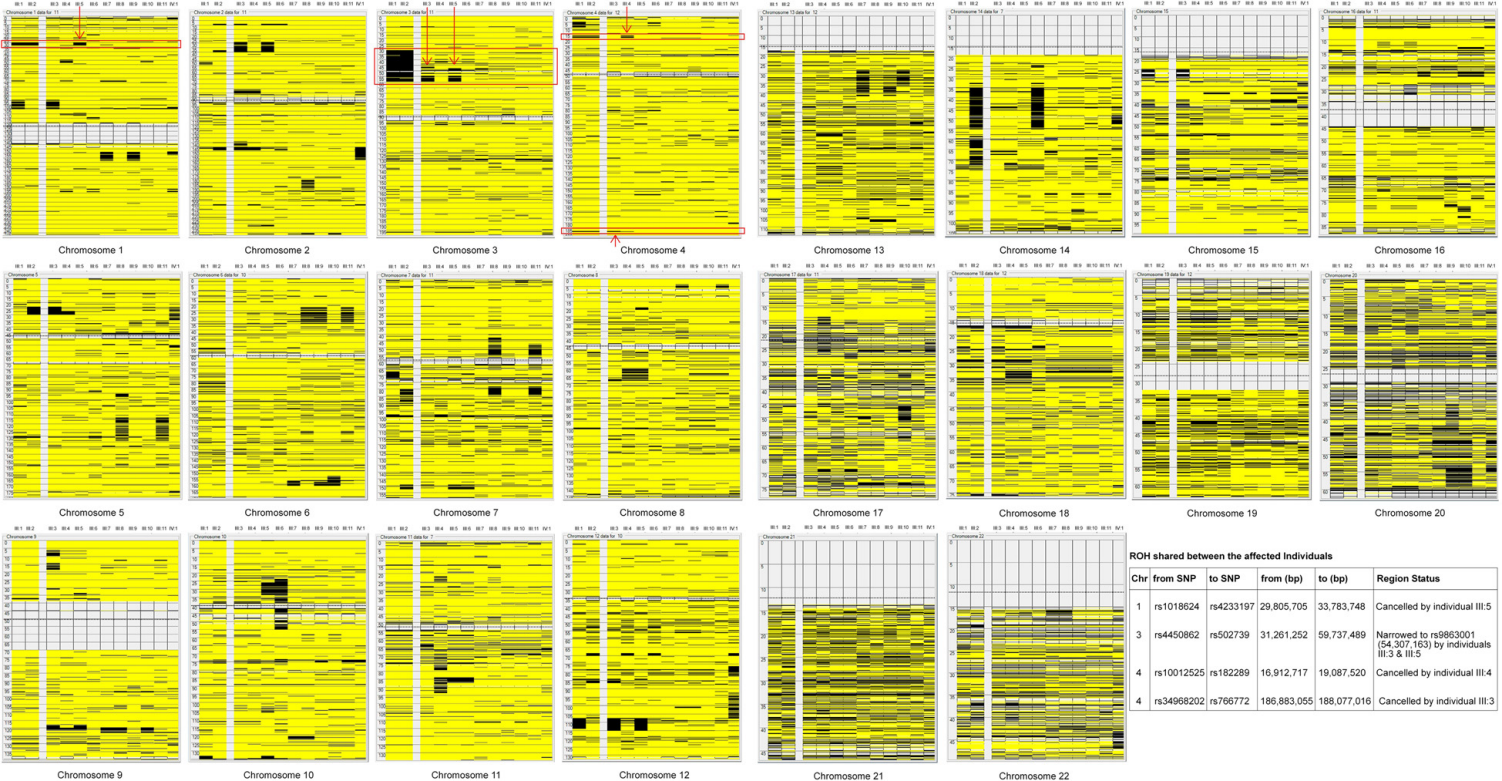
**Detailed AutoSNPa homozygosity mapping analysis for all chromosomes in 12 family members.** The four shared ROH’s in the affected sisters are indicated by red boxes, whose coordinates are detailed in the table. The arrows point to the unaffected individuals that cancel out three of the ROHs on chromosomes 1 and 4. In addition, narrowing of the remaining ROH on chromosome 3 containing *DNAH1* is indicated by red arrows due to shared region present in 2 unaffected siblings (III:3 and III:5)*.*

According to Homozygosity Mapper, the proband and her affected sister shared 4 distinct regions of homozygosity (ROH) the largest being on chromosome 3p21.31-p21 (Figure 1D). Further analysis using AutoSNPa corroborated these results for the 4 ROH but three of these were cancelled out due to unaffected members of the family being homozygous in the same regions and further narrowed down the remaining single region (Figure 1E and Figure 2). This single region, chr3:31,261,252-54,307,163 (hg19 assembly Human Browser Gateway: https://genome.ucsc.edu/cgi-bin/hgGateway?db=hg19) harbored the *DNAH1* gene (RefSeq NM_015512.4) encoding the dynein axonemal heavy chain 1 protein that was initially sequenced in genomic DNA in all 12 family members as a primary candidate.

Sequence analysis of *DNAH1* identified a novel homozygous missense variation in exon 20 (g.52387629A > C; c.3460 A > C; p.Lys1154Gln) present in both affected sisters (Figure 1F) that completely segregated with the disease phenotype (Figure 1C). The variant was not found in 600 ethnically matched normal chromosomes. Protein sequence alignment of DNAH1 orthologs demonstrated that the lysine residue is highly conserved across species (Figure 1G). In addition, p.Lys1154Gln is predicted to be probably damaging with a score of 0.998 on Polyphen (http://genetics.bwh.harvard.edu/pph2/index.shtml), damaging (0.05) using SIFT (Sorting Intolerant from Tolerant; http://sift.jcvi.org/www/SIFT_enst_submit.html) and disease-causing (0.999) using Mutation Taster (http://www.mutationtaster.org/).

### Exome sequencing analysis

There were no causative pathogenic mutations detected in the genes described in Gene panel version DGD_15112013. In exome-wide analysis on the proband the p.Lys1154Gln in *DNAH1* was detected as the only possibly causative variant (Additional file 3: Table S3).

## Discussion

In this case report, using a combination of homozygosity mapping and whole exome sequencing methodology, we describe the identification of a novel variation (c.3460 A > C; p.Lys1154Gln) in the *DNAH1* gene in PCD affected sisters born from a consanguineous union. In addition to the results described here, a previous study described that the phenotype of the *DNAH1* mouse homolog *MDHC7* knockout resulted in asthenozoospermia (decrease in the motility of the spermatozoa) and reduced tracheal ciliary beat frequency using a photo-electrical method [15] and by a study by Blouin and colleagues who identified a potential novel PCD locus on chromosome 3p21 [25] both indicative that aberrations in *DNAH1* may cause PCD. Tissue expression studies showed that DNAH1 is heavily expressed in the testis and trachea, both of which contain axonemal structures that are altered in PCD patients [26]. The lysine residue altered in these patients is located in the highly conserved *N*-terminal stem of the DNAH1 protein. This component is known to interact with other heavy chains and additional essential subunits of the axonemal complex [27]. Hence, the substitution of a basic, strongly positive lysine to a polar uncharged glutamine residue may alter the 3D confirmation and stability of the protein structure; disrupt interaction with other dynein chains and arm subunits and in the function of this heavy chain with regards to ATP hydrolysis used in ciliary and flagellar motility. As mentioned previously [17], variants in *DNAH1* have been reported to cause asthenozoospermia without any accompanying PCD symptoms. They suggested that DNAH1 function in ciliated cells is probably compensated by other HC dyneins, such as *DNAH12* (MIM 603340) as it is the closest paralog. However, the authors noted that none of the parents of their subjects could be analyzed to confirm transmission and hence segregation of the identified variants nor could they exclude the possibility that some of the variants might be hemizygous with a deletion on the other allele. Furthermore, they could not obtain biological samples from their subjects to exclude a reduction of ciliary beats that was seen in the mouse knockout and proposed that future work would require them to perform thorough physical analysis of their male subjects to indicate whether their patients might be at risk of developing late onset PCD. It is pertinent to note that the patients in this study were female and all of the subjects in Ben Khalifa and colleagues were male, possibly suggesting for now that for unknown reasons, females may be more sensitive to a more severe phenotype and/or that the p.Lys1154Gln variant may have a more severe affect than the variants identified in the first study. In addition, the authors cannot exclude the probability that although this variant was the only one found and may play a role in PCD, it is possible that a pathogenic causal variant may be located in an uncharacterized gene or in a known gene that may have missed by the constraints of the methodology used in this study. Further investigation including functional analysis is necessary to determine the effect of this variant on the protein and to explore the presence of SNPs or molecular variants working in parallel with the p.Lys1154Gln variant causing the expanded phenotype seen in the patient described here. With regards to our proband, IVF was successful and pre-implantation genetic diagnosis was cancelled as genotyping of the husband indicated that he was wild-type normal (Figure 1C) and hence all offspring would be obligate carriers. The pregnancy continued uneventfully until term and the proband delivered a healthy baby boy by cesarean section.

## Conclusion

In summary, we have identified PCD patients from the same family with a missense variation that segregates with the disease phenotype in the *DNAH1* gene. In addition to adding *DNAH1* as a gene that may be involved in giving rise to PCD, the clinical diagnosis and the subsequent genetic findings have translated into an overall positive and beneficial outcome for the index patient and family and will be of benefit for future preventative and counseling measures in the future.

### Consent

Written informed consent was obtained from the patient for publication of this case report and any accompanying images. A copy of the written consent is available for review by the Editor of this journal.

## Additional files

Additional file 1: Table S1. Primers used to amplify the coding exons of the *DNAH1* gene. Additional file 2: Table S2. List of genes that are present on the Mendelian-inherited disorders (gene panel version DGD_15112013). Additional file 3: Table S3. A table showing all of the potential homozygous deleterious variants identified by exome sequencing in the proband. The variants identified in chromosome 3 are highlighted in yellow. The p.Lys1154Gln change is highlighted in red and was reported as the likely variant in the patient after numerous filtering and analysis criteria had been applied.
