# Supplementary material for: Variation in DNAH1 may contribute to primary ciliary dyskinesia
Source: BMC Med Genet. 2015 Mar 17;16:14. doi: 10.1186/s12881-015-0162-5 (PMC4422061; doi:10.1186/s12881-015-0162-5)
Supplement: Additional file 1: Table S1. — Primers used to amplify the coding exons of the DNAH1 gene. [file 12881_2015_162_MOESM1_ESM.docx]

**Table S1: Primers used to amplify the coding exons of the *DNAH1* gene.**

|  | **Forward Primer (5’-3’)** | **Reverse Primer (5’-3’)** |
| --- | --- | --- |
| ***DNAH1_exon_*2** | **ACTGACGCTGGGTTCTTCTC** | **ATGGGGAGAAGGATGAAAGG** |
| ***DNAH1_exon_*3** | **GGTCCTGCCTGTCCCTG** | **gaaaaggctgtctccctgag** |
| ***DNAH1_exon_*4** | **GCTTTGAGCTTCCCACACTC** | **ACAGGCTCTGCCATGAGG** |
| ***DNAH1_exon_*5** | **GTAGGCCCTTGTGGACACC** | **ACTGGGGTCCTGCACTTCTG** |
| ***DNAH1_exon_*6** | **CTTTGGCACACCCAGTCC** | **GTGACAGGGCTGCTACTGG** |
| ***DNAH1_exon_*7** | **ACTCAGAGGCCCCTTCCTG** | **GGAGTTGGCCTCAGTCACAG** |
| ***DNAH1_exon_*8** | **cggaacctagtgtttcagcc** | **GGGAAGGTTCCCTGAAGAAG** |
| ***DNAH1_exon_*9** | **GGGTTCACAGGGTGTGGAG** | **AGACAATCCTGGAGGGGAAG** |
| ***DNAH1_exon_*10** | **AAGCACCCTGTGGATCTGTC** | **AGTTCCAAGGGTCTGCCC** |
| ***DNAH1_exon_*11** | **CAGCTATAGGTCGTGGGTCC** | **CCTGGTCCCTGACCCTG** |
| ***DNAH1_exon_*12** | **TGCTCCTGCACACAGGC** | **TGAGCTCAGGGAGCCATC** |
| ***DNAH1_exon_*13** | **CTCCCCATCTCCCCTCTG** | **GCAGGGGATACACATGCAC** |
| ***DNAH1_exon_*14** | **TTGTGCATGTGTATCCCCTG** | **CTGCTGTGGTGTGTGCTG** |
| ***DNAH1_exon_*15** | **GGAAGAGCAGGTGAGGGAAG** | **GCCCTCGGACTCTTAACACC** |
| ***DNAH1_exon_*16** | **AGGTTCCGATTACCCACCAC** | **CTCTGCAGCTCAGCAGCTC** |
| ***DNAH1_exon_*17** | **TGCCACTCCACCACTTCAG** | **GAGAACTGTGCGTGCGTG** |
| ***DNAH1_exon_*18** | **AAGGCCTGGACACAAGTGG** | **GCATCTACATGAGCTGCTTCC** |
| ***DNAH1_exon_*19** | **CAACATGGAGAGAGACTGGG** | **GCCGGGTAAGACAGAAACG** |
| ***DNAH1_exon_*20** | **CTCCTCCCTGCCTCTGC** | **AGTGGCTGTGGTTGCCAG** |
| ***DNAH1_exon_*21** | **TCAGACAGCATCAGGACCAG** | **CGGAGACCATCAGTGACACC** |
| ***DNAH1_exon_*22** | **GACAAACCCCAGCTTGGAC** | **AGGCTACCAGCAATGTGACC** |
| ***DNAH1_exon_*23-24** | **CTGGGATGAGCCTATCTTGC** | **GTTCTCCCTGGGAAGATGC** |
| ***DNAH1_exon_*25** | **TTAGCGCTGGGGCTGTG** | **GGCACTGAGAGGGAGCAG** |
| ***DNAH1_exon_*26** | **AAGAAAGAATGGGATTGGGG** | **GCCCTCCTGTGCCATGTAG** |
| ***DNAH1_exon_*27** | **CTCTGTGAAAGGGGAGGGTG** | **TGACTTTTCTCTTTTCCCCTG** |
| ***DNAH1_exon_*28** | **TATACCCTGCCCAGTGGC** | **TGGGCCAGAGAGAACTAGAGG** |
| ***DNAH1_exon_*29** | **AGGAAATTCCAAGGAAAGGG** | **CCCAAGGAGAGCTAGGCTG** |
| ***DNAH1_exon_*30** | **CCCAGATTGGGCTCTGAAC** | **AGTATGGCGGCGTGAGTATG** |
| ***DNAH1_exon_*31** | **ACTCAGAGGAGGGGACAAGG** | **TGCATAAGTGGCTTAGCATCTC** |
| ***DNAH1_exon_*32** | **AGTCCCAGCGTGTTAGGGAG** | **gtaaagcaccgggcatagag** |
| ***DNAH1_exon_*33-34** | **gggaggcagagtgttccag** | **CCTGGACTCCAAGGTGGC** |
| ***DNAH1_exon_*35-36** | **gtgtggccaggacccaag** | **CAGCCCACAGCCTCCAG** |
| ***DNAH1_exon_*37** | **AAAGTAGAGCCCGCCCAC** | **CTGAGAGAAAGGGGTGAGGG** |
| ***DNAH1_exon_*38-39** | **CTGCAGCCCTTTCTCCAG** | **AGGAGAGCAGTGTGGGGAG** |
| ***DNAH1_exon_*40-41** | **CTCAGGCCGCTTGATACTG** | **TAATTCATTGCGGGAACAGC** |
| ***DNAH1_exon_*42-43** | **AGGAGTGGGGCAGGGAG** | **TAATTTGGTGGAGAGGTGGG** |
| ***DNAH1_exon_*44** | **TGGATTCTCAGAGGACCTGG** | **taggccttcagtgctgtgc** |
| ***DNAH1_exon_*45** | **ggccacaaaggtcataatgc** | **gtccttGGTGTGGCCTCC** |
| ***DNAH1_exon_*46** | **GTGAGCAGCAAGAGGTCCAC** | **CCTCAACACACCCAACCAG** |
| ***DNAH1_exon_*47** | **CAAAGGCAAGGAGGTCAGG** | **GGGGAGGATGCGGAGAG** |
| ***DNAH1_exon_*48** | **CTTGCCTGGTGGTTTGAGAG** | **CAGGGAGGCAGATTGAAGAG** |
| ***DNAH1_exon_*49** | **gaatcggggagaccctacaG** | **AAGCATGGGTCAGTCAAACC** |
| ***DNAH1_exon_*50** | **gtcaCCCACCACCCTTCAG** | **GATGCTATGCTCAGATGGGG** |
| ***DNAH1_exon_*51** | **AGCTGTTGTGAAGGTCCAGC** | **CCATGTACACACCCAGCTCC** |
| ***DNAH1_exon_*52** | **TCCTGGGGTCGTTGGTC** | **atgcccCAAGCTAAACAGG** |
| ***DNAH1_exon_*53** | **CCCAGTCCCTGTCTTTCCTG** | **TGGAGTCAGGGCTGAGTGAG** |
| ***DNAH1_exon_*54** | **CTGTGCTCTCTCTGTCCCTG** | **GGTGCCGAGCTTCCTCAG** |
| ***DNAH1_exon_*55** | **tctggggagactaagatgcag** | **GGATGTGCAGAGGGAAGG** |
| ***DNAH1_exon_*56** | **CCTTTCTGGCAGGGTCC** | **TTCACTGTCCTGCCGGG** |
| ***DNAH1_exon_*57** | **CTTCCCCTGTCACCCTTGAG** | **ATTAGCTCGCCAAGTACCCC** |
| ***DNAH1_exon_*58** | **CTGAGTGGAGCTGGTGGG** | **TGGGAGGCTGGGCTAGG** |
| ***DNAH1_exon_*59** | **ACAGGGGCCAGAAAGGAC** | **GAAGGGTCTCTTTGGGAAGG** |
| ***DNAH1_exon_*60** | **GGGAGGTCTCTGTGAGTGTC** | **GGTAGGAAGGAGGCAGAAGG** |
| ***DNAH1_exon_*61-62** | **GATGCTGATGCCCTCAGTG** | **CAGGGGAAGGGGACTGC** |
| ***DNAH1_exon_*63** | **CTGCCCCACTGGTGATG** | **GGAGAGGAGCAGCTGGG** |
| ***DNAH1_exon_*64** | **CAGGTTCACGACTAGCCCTC** | **CAGGCAGGGAGAGGTGG** |
| ***DNAH1_exon_*65** | **CCACCTCTCCCTGCCTG** | **GTGAGTTTGTGTGTGCCCC** |
| ***DNAH1_exon_*66** | **CGGGGCTCTTCCTTCCTC** | **TCCTTCCACCCTGTTCTCAG** |
| ***DNAH1_exon_*67** | **CAGAGCAGGAGGAGCTGG** | **TTTCCTAGGGACTCATCCCC** |
| ***DNAH1_exon_*68** | **CACCAACCTCCTTCCAACAG** | **TCTATCCAGATGCAGCCAGG** |
| ***DNAH1_exon_*69-70** | **CTTGCCCCGATCTCTCTG** | **gcctcaTTAGTCCCTGATGG** |
| ***DNAH1_exon_*71-72** | **GAGCCTGGTGTCAGGGTG** | **AGGACCTATGGGGTGGGTG** |
| ***DNAH1_exon_*73** | **CCCTACAAGGTGGGCCTG** | **GGTACCCCAGAACCCCAG** |
| ***DNAH1_exon_*74-75** | **TCCATTGGAGGGTAACTCTG** | **CATTTCTCTACAGTCCAGTGGC** |
| ***DNAH1_exon_*76** | **TAGCTACTGCCACGTGACCC** | **CTCAGAGCCTCTCCTCCCTG** |
| ***DNAH1_exon_*77** | **TCAGAAGGGAGTTTTGTGCC** | **TGGGCTGGGTTAGTCCTG** |
| ***DNAH1_exon_*78** | **GCCCCTACGCTATCCCTG** | **GCAAGCAGCTAAGGCACAG** |
