# Supplementary material for: Variation in DNAH1 may contribute to primary ciliary dyskinesia
Source: BMC Med Genet. 2015 Mar 17;16:14. doi: 10.1186/s12881-015-0162-5 (PMC4422061; doi:10.1186/s12881-015-0162-5)
Supplement: Additional file 2: Table S2. — List of genes that are present on the Mendelian-inherited disorders (gene panel version DGD_15112013). [file 12881_2015_162_MOESM2_ESM.docx]

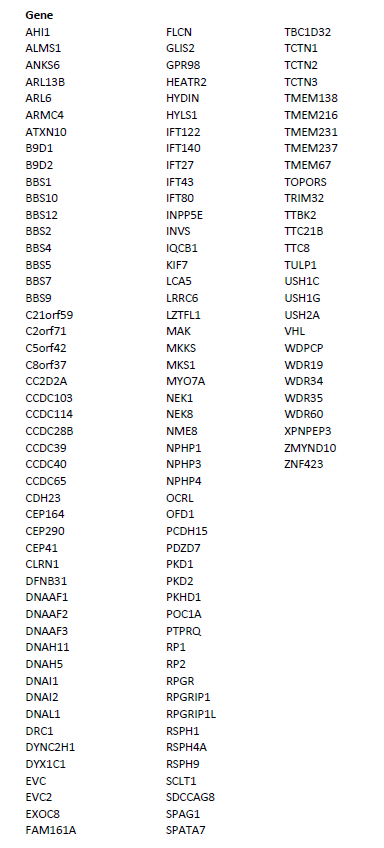


**Table S2: List of genes that are present on the Mendelian-inherited disorders (gene panel version DGD_15112013).**
