# Supplementary material for: Variation in DNAH1 may contribute to primary ciliary dyskinesia
Source: BMC Med Genet. 2015 Mar 17;16:14. doi: 10.1186/s12881-015-0162-5 (PMC4422061; doi:10.1186/s12881-015-0162-5)
Supplement: Additional file 3: Table S3. — A table showing all of the potential homozygous deleterious variants identified by exome sequencing in the proband. The variants identified in chromosome 3 are highlighted in yellow. The p.Lys1154Gln change is highlighted in red and was reported as the likely variant in the patient after numerous filtering and analysis criteria had been applied. [file 12881_2015_162_MOESM3_ESM.docx]

**Table S3: A table showing all of the potential homozygous deleterious variants identified by exome sequencing in the proband. The variants identified in chromosome 3 are highlighted in yellow. The p.Lys1154Gln change is highlighted in red and was reported as the likely variant in the patient after numerous filtering and analysis criteria had been applied.**
